# Controlling the Nanoscale Patterning of AuNPs on Silicon Surfaces

**DOI:** 10.3390/nano3010192

**Published:** 2013-03-21

**Authors:** Sophie E. Williams, Philip R. Davies, Jenna L. Bowen, Chris J. Allender

**Affiliations:** 1 Cardiff School of Pharmacy and Pharmaceutical Sciences, Cardiff University, Cardiff, CF10 3NB, UK; E-Mails: williamsse10@cf.ac.uk (S.E.W.); bowenjl2@cf.ac.uk (J.L.B.); 2 School of Chemistry, Cardiff Catalysis Institute, Cardiff University, Cardiff, CF10 3AT, UK; E-Mail: daviespr@cf.ac.uk

**Keywords:** vapour-phase deposition, APTES, gold, nanoparticle, chemical nanopatterning

## Abstract

This study evaluates the effectiveness of vapour-phase deposition for creating sub-monolayer coverage of aminopropyl triethoxysilane (APTES) on silicon in order to exert control over subsequent gold nanoparticle deposition. Surface coverage was evaluated indirectly by observing the extent to which gold nanoparticles (AuNPs) deposited onto the modified silicon surface. By varying the distance of the silicon wafer from the APTES source and concentration of APTES in the evaporating media, control over subsequent gold nanoparticle deposition was achievable to an extent. Fine control over AuNP deposition (AuNPs/μm^2^) however, was best achieved by adjusting the ionic concentration of the AuNP-depositing solution. Furthermore it was demonstrated that although APTES was fully removed from the silicon surface following four hours incubation in water, the gold nanoparticle-amino surface complex was stable under the same conditions. Atomic force microscopy (AFM) and X-ray photoelectron spectroscopy (XPS) were used to study these affects.

## 1. Introduction

Fabrication and manipulation of nano-sized features is a fast-growing science playing an important role in the development of electronics, materials and biotechnology [1]. Whilst conventional surface patterning methods, such as photolithography and microcontact printing, are restricted to surface patterning in the micrometer range, electron beam lithography can etch materials with line-widths as low as 5 nm [2] whilst scanning probe techniques are able to manipulate structures at the molecular and even atomic scale [3]. Many approaches utilize the nanoscale dimensions of an atomic force microscope (AFM) tip, either to etch a protective layer (anodic oxidation), create electric fields (charge writing) or deposit molecules directly (dip-pen nanolithography and nanofountain probe) onto selected areas [4,5,6]. Whilst AFM techniques are able to pattern very small surface areas, gravure printing has been shown to be able to pattern much larger areas [7]. These “top-down” approaches however, often involve the use of hazardous chemicals, sophisticated equipment and are expensive. Simple molecular self-assembly, for example nanosphere lithography [8], is an alternative and accessible alternative to these approaches.

The aim of this work was to evaluate the effectiveness of a vapour phase deposition protocol for preparing sub-monolayers of aminopropyl triethoxysilane (APTES) on silicon surfaces to control gold nanosphere surface densities. The effect of changing vapour phase deposition parameters on resulting APTES density and the propensity of the modified surface to adsorb gold nanoparticles (AuNPs), have been systematically evaluated. Additionally, the effect of varying the ionic concentration of the incubating AuNP solution on nanoparticle deposition and the protection of underlying chemical functionality conferred by the nanoparticles was investigated. The methods described are simple and practical, allowing reproducible deposition of various AuNP densities and may find application in the patterning of a range of substrates that are amenable to silanisation, including mica, quartz and glass.

## 2. Results and Discussion

AuNPs stabilised with anionic citrate ions bind electrostatically to cationic APTES molecules and have widely been used to confirm the presence of APTES on derivatized silicon wafer [9,10,11]. The effect of evaporative distance, APTES concentration (as first proposed by Bhat *et al.* [11]) and several other experimental variables, on the deposition of gold nanoparticles on a surface, was investigated. The results obtained with variations in experimental conditions are outlined in the following sections:

### 2.1. Controlling the Deposition through Modifying Amine Surface Coverage

#### 2.1.1. Evaporative Distance

**Figure 1:**
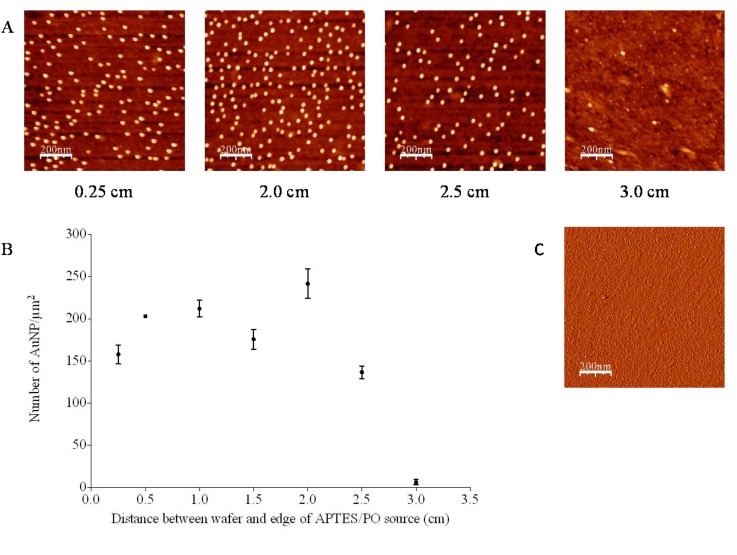
(**A**) Atomic force microscopy (AFM) trace images (after WSXM software processing, *z*-axis = 20 nm, scan size = 1 μm^2^) and (**B**) average gold nanoparticle (AuNP) number density as a function of evaporative distance following immersion in AuNP solution(10 nm, ionic concentration: 7.7 × 10^−4^ M, *n* = 3 ± SD). All surfaces were silanised for 5 min; (**C**) Aminopropyl triethoxysilane (APTES) modified surface prior to AuNP adsorption.

The effect of evaporative distance on the patterning of AuNPs was investigated using 260 μL APTES (50% *w*/*w* in paraffin oil (PO)) as the evaporative solution and with silicon surfaces centered at 0.25, 0.5, 1.0, 1.5, 2.0, 2.5 and 3.0 cm from the edge of the Eppendorf lid. AFM images of the surfaces following immersion in AuNP solution (10 nm, ionic concentration: 7.7 × 10^−4^ M) are shown in Figure 1A along with the average number density for each surface (Figure 1B). Although some variability was noted in the data, in general, the AuNP number density was consistent between 0.25 and 2.0 cm from the APTES source. A sharp decrease was subsequently observed when the evaporative distance was increased to 2.5 cm and again at 3.0 cm when virtually no AuNP deposition was observed. Using a similar method, Bhat *et al*. [11] reported a gradual positive number density gradient from 0.5 to 2.5 cm followed by a sharp drop to ~3.2 cm, with a maximum number density of 480/μm^2^. In this current study the maximum observed number density was 242 ± 17 AuNPs/μm^2^. This discrepancy in AuNP number density is more than likely attributable to a difference in the particle size and ionic concentrations of the AuNP solutions used in the studies. Surfaces positioned up to 3.0 cm (Figure 1C) from the APTES solution showed complete coverage of AuNPs when incubated in an AuNP solution of an ionic concentration of 1.55 × 10^−3^ M, demonstrating a near continuous underlying silane layer. The relative height of the wafers to the lip of the Eppendorf lid containing APTES/PO also influenced the deposition of the AuNPs; when wafers were placed directly on the base of the petri dish, a shadow effect was observed with maximal AuNP number density noted at 1.0 cm with a steady decline in coverage between 1.0 and 2.0 cm. This was not witnessed when the wafers were placed level with the lip of the APTES/PO Eppendorf lid.

#### 2.1.2. APTES Concentration

**Figure 2:**
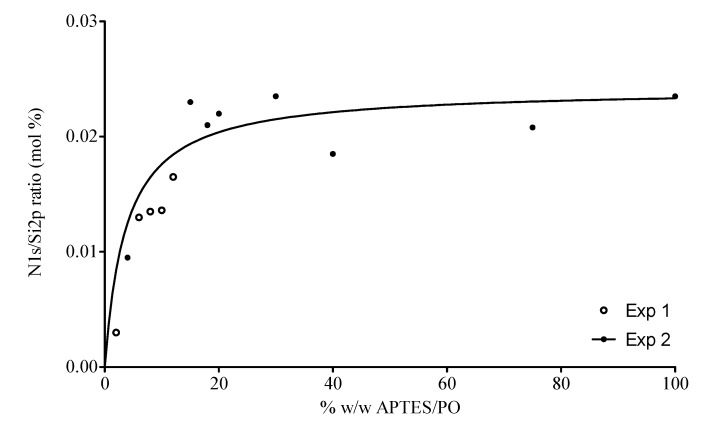
X-ray photoelectron spectroscopy (XPS) analysis of surfaces silanised with varying APTES concentrations. All surfaces were positioned 2 cm from the APTES source and left in place for 5 min. The XPS N1s signal (corresponding to surface-deposited APTES) was used to create a ratio with the Si2p signal (corresponding to APTES and the silicon surface) and plotted for each concentration (*n* = 1).

The effect of APTES concentration on deposition of amino groups on the silicon wafers was investigated by XPS analysis of wafers following evaporation of a range of APTES solutions (2% to 100% *w*/*w* APTES/PO). A clear gradient in coverage (represented as nitrogen content) was observed as the APTES concentration increased from 2% to 20%. Further increasing the concentration had no effect on nitrogen concentration suggesting that monolayer coverage was achieved at 20% APTES concentration. There was no evidence for multilayer coverage since there was no significant increase in surface nitrogen at APTES concentrations >20% (Figure 2).

**Figure 3:**
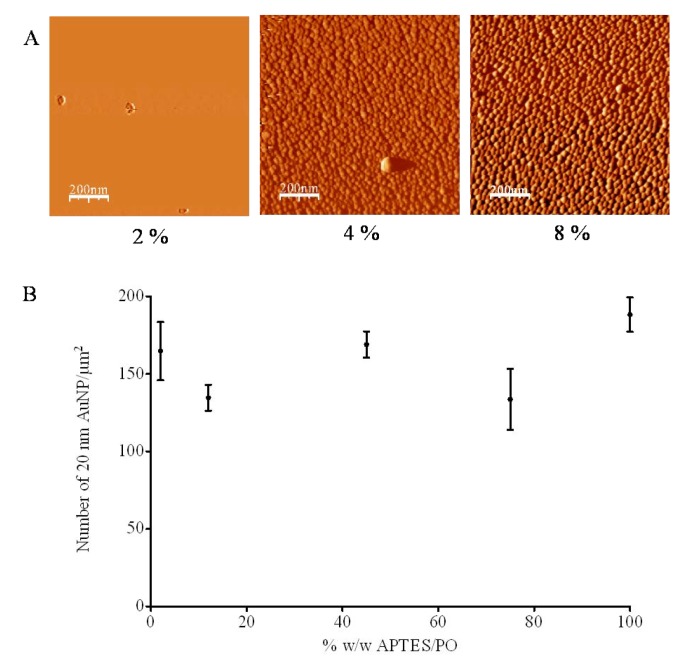
(**A**) AFM trace images (after WSXM software processing, *z*-axis = 20 nm, scan size = 1 μm^2^) of surfaces silanised with 2%, 4% and 8% APTES (silanisation time = 5 min, distance of wafer from source = 2 cm) and immersed in 10 nm AuNP solution and (**B**) average AuNP number density as a function of APTES concentration following immersion in 20 nm AuNP solution (*n* = 3 ± SD).

To evaluate the potential for varying the APTES concentration and hence amine coverage of the silicon wafers to control AuNP deposition, the surfaces analysed by XPS were also incubated in an AuNP solution (ionic concentration: 1.55 × 10^−3^ M). Following immersion in 10 nm AuNP solution, no AuNP deposition was observed on the surfaces silanised with 2% APTES but almost complete coverage was seen at 4% and 8% (Figure 3A). However, when the APTES modified surfaces were incubated in 20 nm, rather than 10 nm, AuNP solution of the same ionic concentration, no significant difference in AuNP surface coverage was observed for wafers silanised with between 2% and 100% APTES solutions (Figure 3B, ~175 AuNPs/μm^2^); the clear gradient observed in the XPS data (Figure 2) was not reflected in AuNP coverage. It has been suggested that this effect is related to the ability of larger particles to span a number of surface APTES groups [12]. The lowest concentration of APTES investigated for vapor-phase derivitisation was 2%, it is possible that lower concentrations would give rise to a more dispersed surface coverage.

### 2.2. Controlling the Deposition through Modifying Exposure to AuNPs

#### 2.2.1. Exposure Time

**Figure 4:**
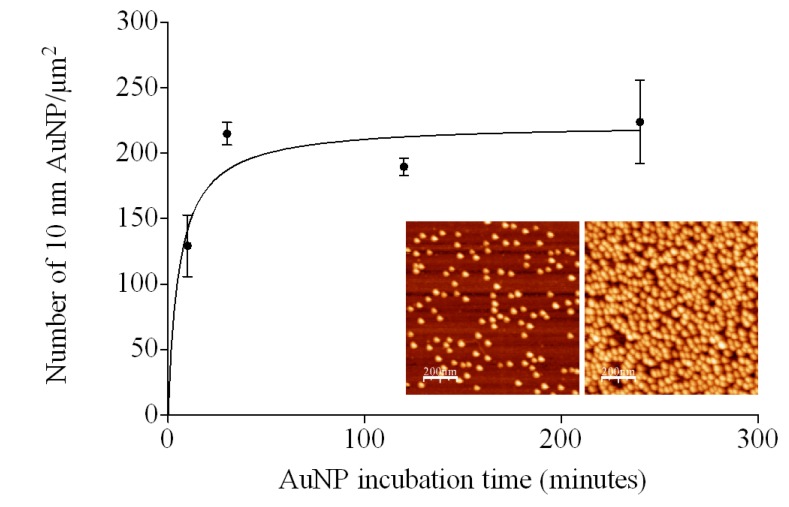
Kinetics of AuNP deposition (10 nm, ionic concentration: 1.55 × 10^−4^ M) on APTES modified silicon wafers (silanisation time = 5 min, distance of wafer from source = 2 cm, *n* = 3 ± SD). Inset: Comparison between APTES modified silicon wafer incubated in 10 nm AuNP solution at two different ionic concentrations for 10 min (**left**: ionic concentration: 1.55 × 10^−4^ M; **right**: ionic concentration: 1.55 × 10^−3^ M).

The ability to control the deposition of AuNPs by varying the length of time the surfaces were exposed to the AuNP solution was investigated. In this kinetic study, silanised surfaces were placed in a 10 nm AuNP solution (ionic concentration: 1.55 × 10^−4^ M) for 10, 30, 120 and 240 min. AuNP surface density reached a maximum (~200 AuNPs/μm^2^) between 10 and 30 min (Figure 4). Higher number densities were achieved at 10 minutes following immersion in a 10 nm AuNP solution of higher ionic strength (ionic concentration: 1.55 × 10^−3^ M) (Figure 4 inset). These results are consistent with a study by Brouwer *et al.* [13] in which it was shown that whilst maximum AuNP surface coverage was directly dependent on ionic concentration, the rate of AuNP deposition was diffusion controlled, limited only by the supply of nanoparticles.

#### 2.2.2. Ionic Strength of the AuNP Solution

The stability of nanocolloidal solutions is based upon repulsion between individual nanoparticles. In aqueous solution, negatively-charged citrate-stabilized AuNPs are surrounded by an electrical double layer of positive ions. Increasing the thickness of the double layer directly increases the electrostatic repulsion and interparticle distance of colloids in solution (Deyagin-Landau and Verwy-Overbeek (DLVO) theory) [14,15,16]. A decrease in ionic strength will increase the double layer thickness around the AuNPs thus increasing the inter-particle repulsion (and hence distances). One would therefore expect to see a decrease in nanoparticle density as the ionic concentration of the AuNP solution decreases. The potential for this phenomenon to control the nanopatterning of silicon surfaces with AuNPs was investigated through the silanisation of surfaces using APTES with subsequent immersion in AuNP solutions of various ionic concentrations (ranging from 1.55 × 10^−3^ to 1.55 × 10^−5^ M).

**Figure 5:**
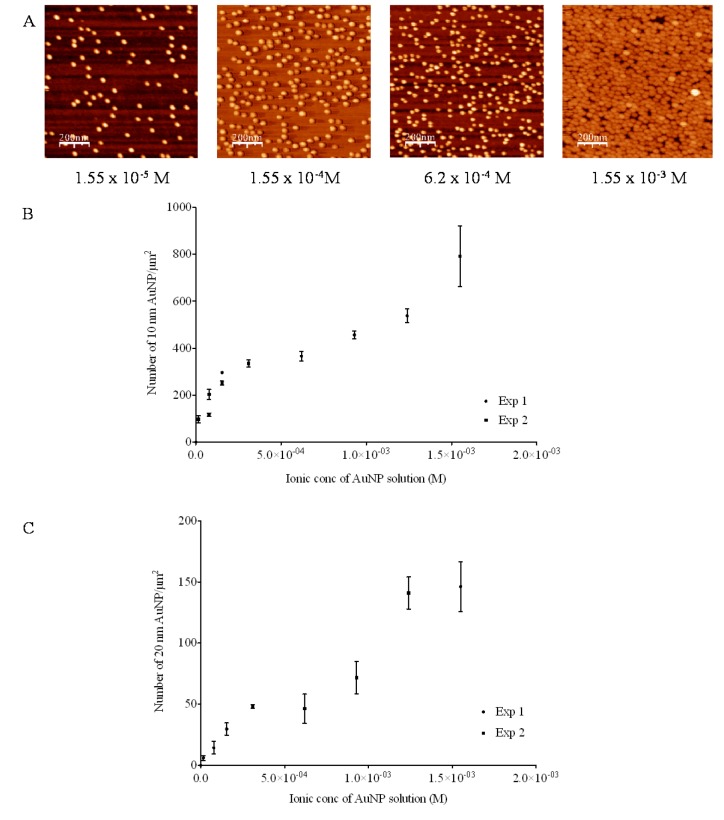
Effect of ionic concentration on surface adsorbed AuNP number density. APTES-derivatised surfaces were incubated in 10 nm AuNP (**A** and **B**) and 20 nm AuNP solutions (**C**) of varying ionic concentrations. The number density (AuNPs/μm^2^) was counted manually from AFM scans (*n* = 3 ± SD). All surfaces were silanised for 5 min at a distance of 2 cm from the APTES source.

The AuNP solutions used in this study were stabilised in a citrate buffer (0.04% trisodium citrate) that was sequentially diluted to give rise to solutions of varying ionic concentration. All solutions, regardless of ionic concentration, were shown to have a pH of between 6.4 and 6.7, *i.e.*, below the immobilized pKa of APTES (immobilized pKa 7.6 [12]). Ionisation will therefore not have influenced the interaction of AuNP with the amine modified silicon surface. Furthermore, in all experiments the number of AuNPs in solution exceeded the number of surface AuNP binding sites. For each solution, the number of AuNPs/mL was calculated from UV absorbance (A_450_) values [17] and multiplied by the volume, to get a total number of nanoparticles in solution. The theoretical number of AuNPs that could bind to the surfaces at maximum packing was calculated using unit cell calculations. In all cases the ratio of nanoparticles in solution: theoretical maximum of surface nanoparticles was >5:1. AFM images plus the average number density of AuNPs for each silicon surface plotted against ionic concentration of the incubating solution for both 10 nm (Figure 5A,B) and 20 nm AuNPs (Figure 5C) are presented. As the ionic concentration of both 10 nm and 20 nm AuNP solutions decreased, the surface density of nanoparticles also decreased. This is attributable to a greater inter-particle repulsion as a consequence of the increasing double layer thickness as described earlier.

### 2.3. Protection of Underlying Amine Groups by AuNPs

**Figure 6:**
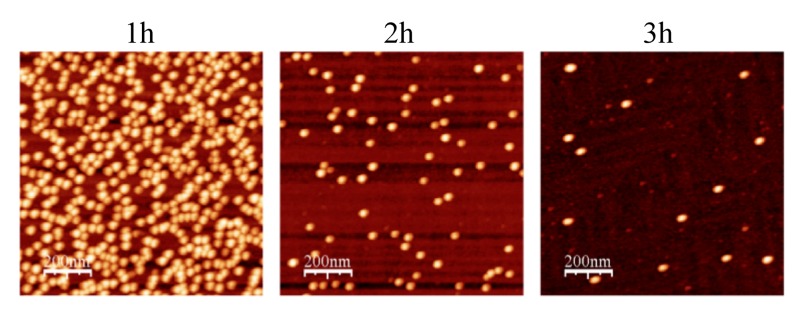
Stability of surface APTES in dH_2_O. Silanised surfaces (silanisation time = 5 min, distance of wafer from APTES source = 2 cm) were placed in 4 mL dH_2_O for 1, 3 and 4 h, followed by immersion in 10nm AuNP solution. A decrease in number density was observed upon incubation of surfaces in dH_2_O, reflecting a decrease in total APTES density (hydrolysis effect). Trace images are shown for each time-point after WSXM software processing (*z*-axis = 20 nm, scan size = 1 μm^2^).

APTES surfaces were incubated for increasing lengths of time in water with subsequent immersion in a solution of AuNPs (ionic concentration: 1.55 × 10^−3^ M). It was observed that on increased exposure to water, a decrease in AuNP surface densities resulted (Figure 6). This is attributed to a decrease in total APTES density as a consequence of the hydrolytic cleavage of silane from the surface [18]. Following 4 h incubation very few AuNPs were bound to the surface. Importantly, this demonstrates that the binding of AuNPs to APTES derivitised silicon occurred much more rapidly than did the removal of APTES from the silicon surface due to hydrolysis. 

**Figure 7:**
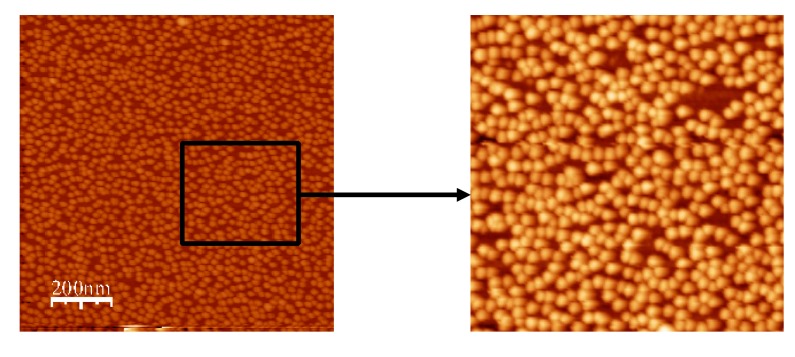
Stability of the APTES-Au interaction. AFM trace image after WSXM software processing (*z*-axis = 20nm, scan size = 1μm^2^. A saturated AuNP surface density was still observed after incubation of an APTES AuNP surface in dH_2_O for 24 h.

Incubation in water following adsorption of AuNPs to silanised surfaces caused no obvious decrease in AuNP number density (Figure 7), demonstrating that the APTES-AuNP interaction was stable and irreversible in water over a period of 24 h thus suggesting that the process of adsorption was not dynamic. 

Incubation of a surface displaying lower AuNP number density in water for 24 h showed no change in AuNP number following re-immersion in an AuNP solution. This confirms the protective effect conferred by the AuNPs on the underlying chemical functionality.

## 3. Experimental Section

### 3.1. Vapor-Phase Silanisation

**Figure 8:**
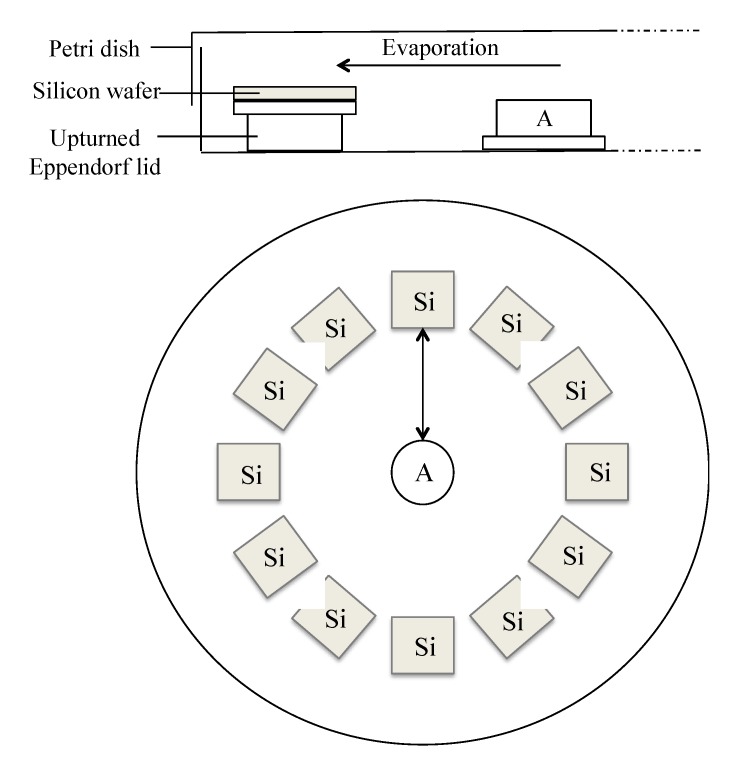
Side-on and birds-eye view of the arrangement of silicon wafers (Si) in a petri dish around an Eppendorf lid containing APTES in paraffin oil (A) during vapor-phase deposition experiments. All silicon wafers were placed equidistant from the APTES source (2 cm).

Thermally oxidised silicon test wafers (polished one side, 600–700 μm thickness, Boron and Phosphorous-doped (Compart Technologies, Peterborough, UK)) were cut into ~0.5 cm^2^ pieces. Glassware was cleaned in 2% DECON (Fisher, Waltham, MA, USA). All experiments were carried out at room temperature and pressure. Surfaces were degreased by ultrasonication in ethanol (15 min) followed by dimethylformamide (15 min), then dried under a stream of nitrogen. Surfaces were submerged in Piranha solution (7 mL:3 mL, H_2_SO_4_:H_2_O_2_) and maintained at 80–90 °C (CAUTION: *piranha solution strongly reacts with organic materials and should be handled with extreme caution*). Surfaces were washed thoroughly in diH_2_O and dried under a stream of nitrogen. Unless otherwise stated, the activated surfaces were placed 2 cm from the edge of an upturned Eppendorf tube lid and enclosed within a Petri dish (Figure 8). A mixture of 3-aminopropyltriethoxysilane (APTES) solution (Sigma-Aldrich, St. Louis, MO, USA) and paraffin oil (PO) was pipetted into the Eppendorf lid and APTES was left to evaporate over the enclosed surfaces for 5 min. The surfaces were ultrasonicated to remove physisorbed silane, twice washed in acetone (15 min), dried under a stream of nitrogen and oven-cured overnight at 80 °C.

### 3.2. AuNP Patterning of APTES Modified Silicon

Unless otherwise stated, surfaces were incubated in AuNP solution (10 nm or 20 nm diameter, stabilised suspension in citrate buffer (Sigma-Aldrich, St. Louis, MO, USA)) for 14–24 h and then rinsed thoroughly in dH_2_O to remove physisorbed AuNPs. The ionic concentration of the solution, with respect to trisodium citrate, is given for each solution.

### 3.3. Atomic Force Microscopy

A Veeco Scanning Probe Microscope (Veeco Instruments Inc., Woodbury, NY, USA) was used in tapping mode for AFsM analysis of the surfaces using RTESP 1–10 Ohm-cm phosphorous (n) doped silicon AFM tips. All consumables were purchased from Veeco Instruments Inc. AFM images were processed using “WSXM” image analysis [19]. To determine the number of AuNPs deposited “2nd order plane-fit” and “flatten offset” tools were used. WSXM “flooding analysis” was used to determine the number density of AuNPs.

### 3.4. X-Ray Photoelectron Spectroscopy

X-ray photoelectron spectroscopy (XPS) was carried out using a Kratos Axis Ultra DLD spectrometer (Kratos Analytical, Shimadzu, Kyoto, Japan). A monochromatic AlKα X-ray source (75–150 W) with an analyzer pass-energy of 160 eV (survey scans) or 40 eV (detailed scans) was used. Photoelectrons were detected in a direction normal to the surface (*i.e.*, 90° grazing angle). Surfaces were mounted using double-sided adhesive tape. The data was analyzed using Casa XPS [20].

## 4. Conclusions

Evaporation of APTES from paraffin oil was shown to produce highly reproducible monolayer coverage of APTES on silicon surfaces. Control over APTES surface concentration was achieved by varying the concentration in the evaporating medium, which was confirmed by XPS analysis, however AuNP surface densities did not reflect this concentration gradient. This has been attributed to the ability of AuNPs to span a number of surface-immobilized groups, therefore, for a given ionic concentration of AuNP solution, the resulting surface AuNP number density remained constant over the range of APTES concentrations investigated in this study. For a given APTES concentration, AuNP number density was dependent on evaporative distance. Control of AuNP adsorption, albeit with a random (but well spaced) distribution, onto silanised surfaces was shown to be readily achievable by varying the ionic concentration of the AuNP solutions. The protective nature of AuNPs on the underlying APTES groups will allow for chemical nanopatterning, through the selective modification of the amine groups not involved in the binding interaction with the nanoparticles, to generate a bi-functional surface.
